# Dissecting maize diversity in lowland South America: genetic structure and geographic distribution models

**DOI:** 10.1186/s12870-016-0874-5

**Published:** 2016-08-26

**Authors:** Mariana Bracco, Jimena Cascales, Julián Cámara Hernández, Lidia Poggio, Alexandra M. Gottlieb, Verónica V. Lia

**Affiliations:** 1 Departamento de Ecología, Genética y Evolución, Facultad de Ciencias Exactas y Naturales, Universidad de Buenos Aires, Intendente Güiraldes y Costanera Norte s/n, 4to, Piso, Pabellón II, Ciudad Universitaria, C1428EHA Ciudad Autónoma de Buenos Aires, Argentina; 2 Escuela de Ciencias Agrarias, Naturales y Ambientales, Universidad Nacional del Noroeste de la Provincia de Buenos Aires, Av. Pte. Dr. Arturo Frondizi, N° 2650 Pergamino, Buenos Aires Argentina; 3 Consejo Nacional de Investigaciones Científicas y Técnicas (CONICET), Avenida Rivadavia 1917, C1033AAJ Ciudad Autónoma de Buenos Aires, Argentina; 4 Facultad de Agronomía, Universidad de Buenos Aires, Av. San Martín 4453, C1417DSE Ciudad Autónoma de Buenos Aires, Argentina; 5 Instituto de Biotecnología, CICVyA, INTA, N. Repetto y De las Cabañas s/n 1686, Hurlingham, Buenos Aires Argentina

**Keywords:** Genetic diversity, Geographic distribution models, Guaraní communities, Lowland South America, Maize landraces

## Abstract

**Background:**

Maize landraces from South America have traditionally been assigned to two main categories: Andean and Tropical Lowland germplasm. However, the genetic structure and affiliations of the lowland gene pools have been difficult to assess due to limited sampling and the lack of comparative analysis. Here, we examined SSR and *Adh2* sequence variation in a diverse sample of maize landraces from lowland middle South America, and performed a comprehensive integrative analysis of population structure and diversity including already published data of archaeological and extant specimens from the Americas. Geographic distribution models were used to explore the relationship between environmental factors and the observed genetic structure.

**Results:**

Bayesian and multivariate analyses of population structure showed the existence of two previously overlooked lowland gene pools associated with Guaraní indigenous communities of middle South America. The singularity of this germplasm was also evidenced by the frequency distribution of microsatellite repeat motifs of the *Adh2* locus and the distinct spatial pattern inferred from geographic distribution models.

**Conclusion:**

Our results challenge the prevailing view that lowland middle South America is just a contact zone between Andean and Tropical Lowland germplasm and highlight the occurrence of a unique, locally adapted gene pool. This information is relevant for the conservation and utilization of maize genetic resources, as well as for a better understanding of environment-genotype associations.

**Electronic supplementary material:**

The online version of this article (doi:10.1186/s12870-016-0874-5) contains supplementary material, which is available to authorized users.

## Background

There is general consensus that maize (*Zea mays* L. ssp. *mays*) was domesticated from its wild relative, the teosinte *Zea mays* ssp. *parviglumis* Iltis & Doebley, in the lowlands of south-western Mexico during the early Holocene, whence it spread rapidly northwards and southwards across America [1–4]. In South America, the earliest evidence of maize can be traced to at least 7000 calibrated years before present [4, 5]. In this region, however, the introduction date and dispersal routes of the cultigen, as well as the patterns of racial diversification, still remain unclear.

Based on the cytogenetic analysis of South American landraces, McClintock et al. [6] suggested that different types of maize were early introduced into two initial centres of cultivation: northern South America and the central Andean highlands. They proposed that maize germplasm from the northern region had a vast influence on the races of the Caribbean Islands and on those in eastern South America, whereas races from the Andean centre spread extensively throughout the southwest. This hypothesis is consistent with the analysis of a microsatellite repeat within the alcohol dehydrogenase 2 gene (*Adh2*) in archaeological maize specimens, which revealed an east–west partitioning of allele frequencies. These studies provided evidence of two separate expansion events in South America; one occurring from the highlands of Central America into the Andean region, and the other from the alluvial regions of Panama into the lowlands and then along the northeast coast of the continent [7].

More recently, Vigouroux et al. [8] inferred an alternative model of maize introduction from the analysis of SSR (Simple Sequence Repeat) variability, based on a comprehensive assembly of landraces from the Americas. Using genetic distance clustering methods, these researchers concluded that maize cultivation was first introduced into South America from Colombia and Venezuela, subsequently into the Caribbean from South America via Trinidad and Tobago, and into the Andes from Colombia.

Knowledge of the structure of genetic variation in present-day maize landraces, and how it is related to their geographical distribution, provides valuable insights for reconstructing dispersal routes. Maize landraces from the highlands of South America (i.e., the Andean region) have emerged repeatedly as a distinct entity, as evidenced by morphological, cytogenetic and molecular data [1, 6, 9, 10]. In contrast, no clear delimitation could be achieved among the landraces from the lowland regions of South America, adding further complexity to the testing of dispersal hypotheses. Over the years, studies of molecular diversity have referred to germplasm from the lowlands of South America by different names, such as Other South American maize [1], the Tropical Lowland group [8], the Lowland South American group [11], the South American Lowland, Bolivian Lowland and Costal Brazil groups [12], and the Middle South American group [13]. However, differences in geographical sampling between studies and the lack of integrative analyses make it difficult to assess whether these assemblages belong to the same gene pool and how they relate to each other.

Recent research concerning the population dynamics of maize landraces has also provided evidence for a highland-lowland genetic structuring pattern. Bracco et al. [14] conducted a comparative evaluation of SSR variability in an extensive sample of maize landraces from Northeastern and Northwestern Argentina (NEA and NWA, respectively) and identified three distinct gene pools named NWA, NEA popcorns, and NEA flours. The affiliation between the NWA group and the Andean complex was already established by Lia et al. [15], whereas the relationships between landraces from lowland NEA and other regions of lowland South America have not been determined until this study.

Regardless of what the most plausible hypothesis of maize dispersal may be, it is undisputed that its spreading was accompanied by a remarkable adaptation to heterogeneous environmental conditions [11, 16, 17]. Some difficulties may arise in modelling the geographic distribution of crop species, such as discriminating the relative contribution of demography, farmer’s selection and habitat suitability. Notwithstanding this, the methods used in geographic distribution modelling may offer a new perspective to understand current and historical patterns of local adaptation. Indeed, these approaches have provided valuable insights into the ecological requirements and possible impacts of climate change on teosintes and Mexican landraces [16, 17].

Herein we provide a comparative framework to analyse maize diversity and clarify the relationships among lowland South American gene pools. For this purpose, the sampling conducted by Bracco et al. [14] (345 individuals from Northern Argentina) was expanded by genotyping 232 individuals of 12 additional lowland landraces from NEA using SSR markers, and these data were analysed in combination with the datasets of Vigouroux et al. [8] and Lia et al. [15]. In addition, *Adh2* microsatellite sequences were obtained from representative individuals of NWA and NEA to allow comparison with the archaeological and extant specimens from southern South America studied by Freitas et al. [7] and Grimaldo Giraldo [18]. Finally, the relationship between bioclimatic variables and the geographical distribution of the inferred gene pools was explored using species distribution modelling approaches.

## Methods

### SSR genotyping

To fully represent the racial diversity of maize in NEA and to complement the data presented by Bracco et al. [14], 10 SSR loci (*bnlg1866, phi037, bnlg1182, bnlg252, bnlg1287, bnlg1732, bnlg1209, bnlg1018, bnlg1070* and *bnlg1360)* were genotyped on 232 individuals from the Argentinean provinces of Chaco, Corrientes, Entre Ríos, Formosa and Misiones (Additional file 1: Figure A1). Landraces were collected in 1977, 1978 and 2005 directly from farmer fields and preserved at the Banco de Germoplasma EEA INTA Pergamino, or at the Laboratorio de Recursos Genéticos Vegetales “N.I. Vavilov”, Universidad de Buenos Aires. Racial identification, voucher specimens, collection sites and sample sizes of the accessions genotyped here are given in Additional file 1: Table A1. Seed germination, DNA extraction, and SSR genotyping were performed as detailed in Bracco et al. [19]. Primer sequences are available at the MaizeGDB website (http://www.maizegdb.org/data_center/locus).

### SSR Data analysis

To put the SSR data in a continental context, our dataset was analysed in combination with those of Bracco et al. [14], Lia et al. [15] and Vigouroux et al. [8], yielding a final data matrix of 10 SSR and 1288 individuals. This compiled SSR data matrix consisted of 709 individuals belonging to 29 landraces from NEA and NWA, plus 579 individuals from the main genetic groups recognised by Vigouroux et al. [8], namely, Tropical Lowland (74 landraces, 187 individuals), Andean (89 landraces, 235 individuals), Highland Mexico (HM) (37 landraces, 87 individuals) and Northern and Southwestern US (US) (49 landraces, 70 individuals). Individuals identified as admixed in the original studies were excluded from the analysis.

Allele size equivalences between maize landraces from NWA and those of Matsuoka et al. [1] were determined by Lia et al. [15]. Individuals from NWA were then used as allele size markers to genotype NEA landraces. The SSR data of Matsuoka et al. [1] are included within the data set of Vigoroux et al. [8], thus allowing the integration of all sources of data. The compiled SSR matrix is given in Additional file 2: Table A2.

### Model-based clustering

Genetic clusters were inferred using the Bayesian approach implemented in STRUCTURE 2.3.4 [20]. However, given the differences between the number of loci genotyped in NEA and NWA landraces and those used by Vigouroux et al. [8], we first checked the consistency of the groups defined by these authors against the reduction of the number of loci (from 84 to 10) using STRUCTURE. As a result, we retrieved the same four groups reported by them, thus validating the use of this subset of SSR in our integrative analysis (data not shown). The overall probability of identity and the probability of identity given the similarity between siblings were estimated across the complete SSR data matrix according to Waits et al. [21], using GeneAlEx 6 [22]. The discriminant power of the selected loci on the combined dataset was supported by a probability of identity of 4.7 × 10^−14^ and probability of identity among siblings of 4.7 × 10^−5^. Analyses were performed using *K* values from 1 to 10, 10 replicate runs *per K* value, a burn-in period length of 10^5^ and a run length of 10^6^. No prior information on the origin of individuals was used to define the clusters. All the analyses were run under the correlated allele frequency model [23]. The run showing the highest posterior probability was considered for each *K* value. A measure of the second order rate of change in the likelihood of *K* (*ΔK*) [24] was calculated using Structure Harvester [25]. An individual was assigned to one of the clusters on the basis of having a membership coefficient higher than an arbitrary cut-off value of 0.80 (Q > 0.80) (Additional file 2: Table A2). Results were plotted with DISTRUCT 1.1 software [26]. The model of correlated allele frequencies of Falush et al. [23] was applied to estimate the drift parameter (F) for the genetic groups inferred by STRUCTURE. This parameter measures the extent of a cluster’s differentiation relative to a hypothetical population of origin and has a direct interpretation as the amount of genetic drift to which the cluster has been subjected [27]. A graphical representation of the densities of F was obtained using the *density* function of R 3.0.2 (R Core Team 2014).

### Discriminant Analysis of Principal Components (DAPC)

Population structure was also examined by the Discriminant Analysis of Principal Components (DAPC) [28], using the adegenet 1.3-1 package [29] implemented in R 2.13.2 (R Core Team 2014). The function *dapc* was executed by retaining 150 principal components, which accounted for 96 % of total genetic variation and five discriminant functions, and using the clusters identified by the K-means algorithm [30]. The number of clusters was assessed using the function *find.clusters*, with n.iter = 1000000 and n.start = 25, evaluating a range from 1 to 40.

### Genetic diversity and differentiation

Diversity indices were calculated for the genetic clusters inferred by Bayesian analysis, using only individuals unequivocally assigned to their respective clusters (Q > 0.80). The mean number of alleles *per* locus (*A*), allelic richness (*R*s) [31] and gene diversity (*H*e) [32] were estimated using the software Fstat 2.9.3.2 [33]. The presence of group-specific alleles (hereafter referred to as private alleles) was examined for each group. Private allelic richness, i.e., the mean number of private alleles per locus as a function of standardised sample size, was computed with ADZE 1.0 [34]. File conversion was conducted with Convert 1.31 [35].

Differentiation between STRUCTURE groups was assessed by the Allele frequency divergence estimate provided by the software. Cluster analysis was carried out applying the Neighbour-joining algorithm [36] implemented in PHYLIP 3.6 [37]. Branch support was estimated by bootstrapping (1000 pseudoreplicates) with Powermarker 3.25 [38]. Resulting trees were visualised with FigTree 1.3.1 [39].

### Adh2 microsatellite analysis

Individuals of 16 landraces from Northern Argentina (NWA + NEA) were sequenced for the *Adh2* microsatellite region (Additional file 1: Table A3). The *Adh2* gene segment employed for primer design (GenBank X02915) included part of the 5’ untranslated promoter region, the exons 1 and 2, and the intervening intron. PCR primers were designed with the Primer3 software 0.4.0 [40]: upstream, 5’-AAAATCCGAGCCTTTCTTCC-3’; downstream, 5’-CTACCTCCACCTCCTCGATG-3’. Cycling was carried out as in Freitas et al. [7], and the PCR products were checked and visualised as in Bracco et al. [14]. To determine whether individuals were homozygous or heterozygous, PCR products were subjected to native PAGE as described in Bracco et al. [19]. Bands from homozygous individuals (ca. 350 bp in length) were excised from agarose gels (2 % w/v) purified using an AccuPrep® Gel purification kit (BIONEER) and sequenced in an ABI 3130XL apparatus (Applied Biosystems). Chromatograms were edited with BioEdit 7.1.3.0 [41]. Both strands were assembled with the same program, and the microsatellite motifs were extracted for frequency calculation. All sequences were deposited in GenBank under accession numbers KU304471 to KU304494.

The data gathered were analysed together with *Adh2* microsatellite alleles derived from the archaeological landraces examined by Goloubinoff et al. [42], Freitas et al. [7] and Grimaldo Giraldo [18], under the assumption that genealogical continuity exists between the archaeological genetic structure and that of extant landraces. In addition, we extracted the SSR alleles from the modern, primitive and historic landraces studied by Freitas et al. [7] and Grimaldo Giraldo [18], and from *Adh2* sequences retrieved from GenBank corresponding to Brazilian and Bolivian landraces (EU119984-9 and EF070151-6, respectively). The compiled data set (*N* = 497) was divided into three allele types following Freitas et al. [7], i.e. (GA)_n_, (GA)_n_TA and GAAA(GA)_n_.

Statistical associations between allele types and geographic regions were evaluated with the likelihood ratio Chi-square test implemented in Infostat software 2013 [43].

### Modelling of geographic distributions

Geographic distributions were modelled using the Maximum Entropy method [44], implemented in MaxEnt 3.3.3.a (https://www.cs.princeton.edu/~schapire/maxent/). This program uses presence-only data in the form of geo-referenced occurrence records, and a set of environmental variables to produce a model of the distribution range of the species under study. The raw and logistic outputs of MaxEnt are monotonically related and can be interpreted as an estimate of habitat suitability [45, 46].

We modelled the geographic distribution of the genetic groups retrieved from Bayesian clustering, considering only individuals with Q > 80 %. Following the guidelines provided in Scheldeman & van Zonneveld [47], a minimum of 20 occurrence points was set as threshold for modelling. Thus, a total of 317 spatially unique records were used, corresponding to the following clusters: 22 for NEA Flours, 92 for Tropical Lowland, 120 for Andean, and 87 for Highland Mexico and US (HM-US). The NEA Popcorns could not be subjected to analysis because of the low number of unique sampling localities (<5). Given the stability of the MaxEnt method in the face of correlated variables [45], and to facilitate comparisons with previous models of the geographic distribution of maize [16], we used the variable Altitude and the 19 bioclimatic variables available at WORLDCLIM 1.4 (http://www.worldclim.org/) [48] at 2.5 arc-min resolution. These 19 variables are derived from monthly temperature and precipitation records, reflecting seasonal and annual variations. Models were generated using 20,000 background points from all over the world. This implies that landrace could have dispersed anywhere in the globe, which is a reasonable assumption for a domesticated crop such as maize. It has been shown that the predictive ability of MaxEnt models is influenced by the choice of feature types and regularisation parameters, particularly for small sample sizes [49]. By default, the program uses class-specific regularisation parameters tuned on the basis of a large international dataset [45]. In addition, when few samples are available, MaxEnt restricts the model to simple feature classes (i.e., linear, quadratic, hinge) [49]. Because the number of occurrences for the different groups ranged from 22 to 118, we followed the recommendations of Elith et al. [45]; thus, we constructed the models under two different settings: 1) hinge features only and default regularisation parameters; and 2) default feature and regularisation parameters. Models were compared using sample-size corrected Akaike’s Information Criteria (AICc) with ENMtools 1.3 [50, 51].

Model performance was assessed using the area under the receiver operating characteristic curve (AUC) [52] for both training and testing data sets. Ten-fold cross-validation was used to estimate errors around fitted functions and predictive performance on held-out data, except for the NEA Flours in which 4-fold cross-validation was used due to the low number of spatially unique records. Variable importance was determined by measuring the contribution of each variable to model improvement during the training process (percent of contribution), and by jackknife tests implemented in MaxEnt. Model predictions were visualised from MaxEnt logistic outputs using DIVA-GIS 7.2.1.1. [53]. Pairwise comparisons of model predictions were carried out by calculating the *I* statistic of Warren et al. [54] and Schoener’s *D* [55] as implemented in ENMtools 1.3. To evaluate whether the models generated for each genetic cluster are more different than expected if they were drawn from the same underlying distribution, we performed the niche identity test [54] included in ENMtools 1.3 with 100 replicates.

All the maps used in this study were freely available at http://www.diva-gis.org/Data.

## Results

### Inference of genetic clusters

**Fig. 1:**
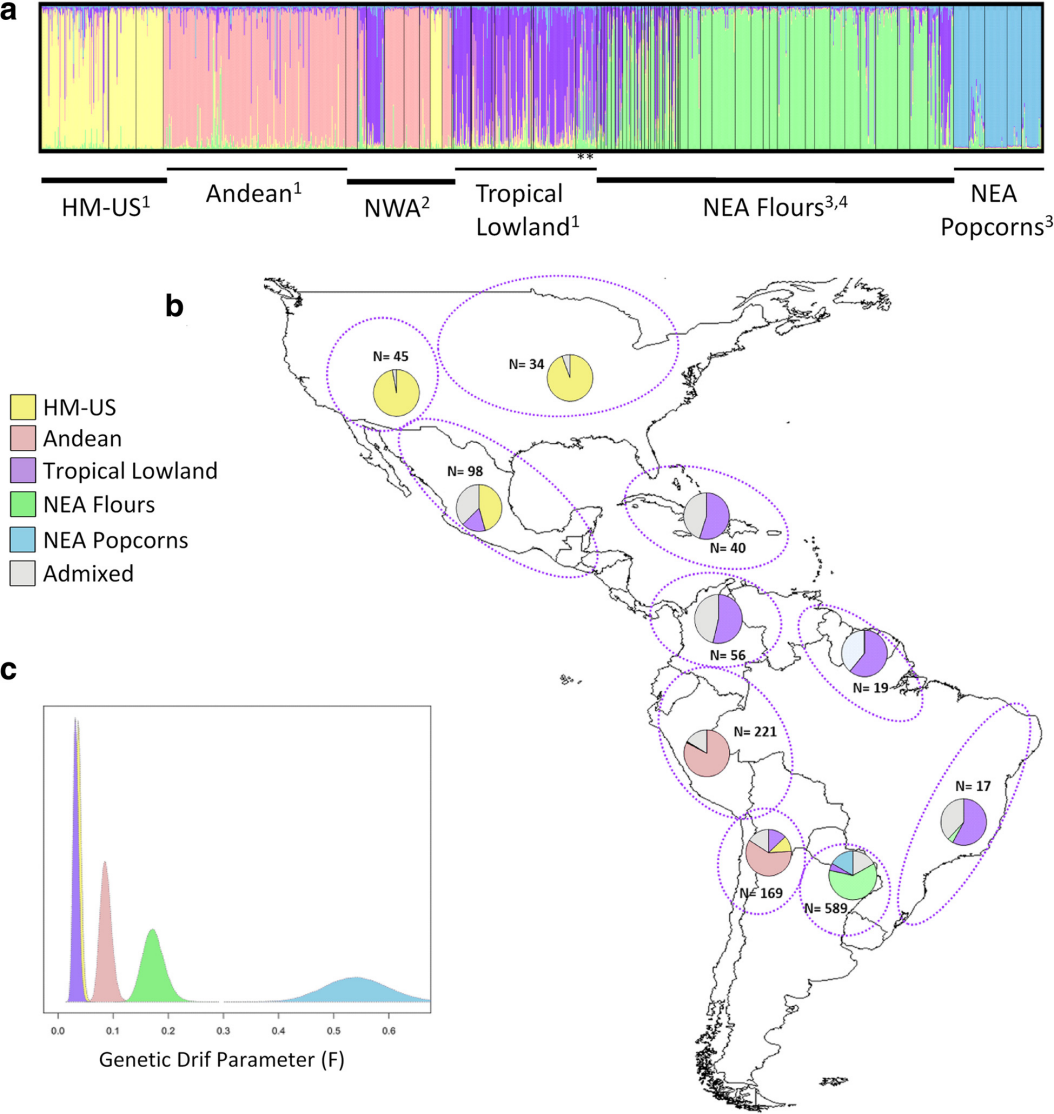
Estimated population structure of maize landraces from the Americas. **a** STRUCTURE bar plots for *K* = 5. Each vertical line represents an individual and colours represent their inferred ancestry from *K* ancestral populations. Individuals are ordered by sampling region and source study. **b** Geographical distribution of the clusters inferred by STRUCTURE. Dotted lines indicate the geographic extent considered for each chart. **c** Posterior densities of the genetic drift parameter (F) from STRUCTURE correlated allele frequency model. ^1^ Data from Vigouroux et al. [8]; ^2^ data from Lia et al. [15]; ^3^ data from Bracco et al. [14]; ^4^ data from this study. * Brazilian accessions. HM-US: Highland Mexico and US; NWA: North Western Argentina; NEA: North Eastern Argentina

Bayesian population structure analysis of the complete SSR data matrix (10 SSR, 1288 individuals) supported the existence of five distinct genetic clusters, as inferred from the joint assessment of the log-likelihood of the data conditional on *K* (LnP(D)) and the rate of change in the log likelihood of the data between successive *K* values (∆K) (Additional file 1: Figure A2). As suggested in previous studies [8], the peak of *∆K* at *K* = 2 was considered an artefact resulting from the low likelihoods of *K* = 1. At *K* = 5, the retrieved clusters showed a clear geographic patterning. We recovered Highland Mexico and US landraces forming a single cluster, (hereafter referred to as HM-US), and two of the main groups previously described for South America by Vigouroux et al. [8], i.e. Andean and Tropical Lowland. The remaining two groups were primarily composed of NEA floury landraces (hereafter referred to as NEA Flours) and NEA popcorn landraces (hereafter referred to as NEA Popcorns) (Fig. 1). To test whether differences in sampling strategies and intensities among the groups could influence STRUCTURE results, we carried out a new STRUCTURE analysis with a reduced number of NEA individuals (a random subset of 50 individuals from NEA Flours and 50 individuals from NEA Popcorns). As a result, we retrieved the same five previously identified groups, suggesting that the observed distinctiveness of NEA landraces was not an artefact due to redundant sampling (Additional file 1: Figure A3).

Analysis of STRUCTURE outputs showed that membership coefficients (Q) in a given cluster were higher than 0.80 for 79 % of the individuals. Following this criterion, 145 individuals were assigned to HM-US, 275 to Andean, 157 to Tropical Lowland, 344 to NEA Flours, and 99 to NEA Popcorns, whereas 268 (21 %) were classified as admixed (Additional file 2: Table A2). Interestingly, accessions from Brazil previously ascribed to the Tropical Lowland group by Vigouroux et al. [8] had a relatively high contribution from the NEA Flours (average Q = 0.25), with some individuals reaching Q ≥0.80. Conversely, several NEA Flours individuals showed remarkable contributions from the Tropical Lowland gene pool, particularly those collected outside the Guaraní communities in the province of Misiones, Argentina (Fig. 1, Additional file 2: Table A2).

The genetic drift parameter identified the Tropical Lowland (mean *F* = 0.032) and HM-US (mean *F* = 0.038) as the most similar to a common hypothetical ancestor, followed by the Andean (mean *F* = 0.087), whereas the NEA Flours (mean *F* = 0.172) and the NEA Popcorns (mean *F* = 0.539) appeared as the most divergent groups (Fig. 1c).

The genetic structuring patterns obtained using DAPC were mostly concordant to those from Bayesian analysis, but group boundaries were less clearly defined. The k-means algorithm identified k = 20 as the most likely number of groups; however, a close inspection of individual assignments showed that many of these groups resulted from the subdivision of the five clusters obtained by STRUCTURE (Additional file 1: Figure A4).

**Fig. 2:**
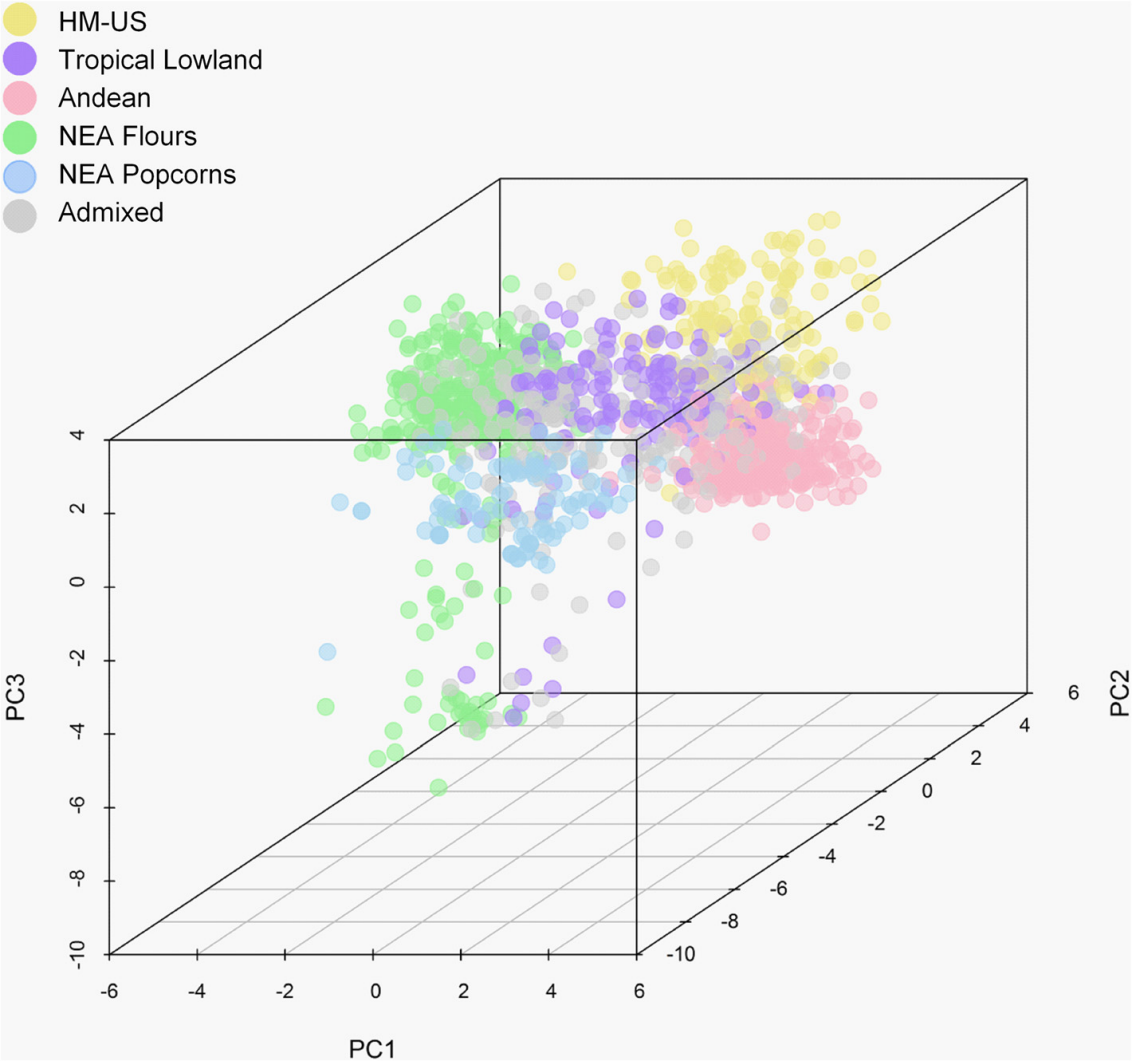
Multivariate analysis of SSR variation in maize landraces from the Americas. Scatterplot of the Discriminant Analysis of Principal Components (DAPC). Dots represent individual samples coloured according to the STRUCTURE assignments. HM-US: Highland Mexico and US

To visually compare the results between analyses, we generated DAPC scatterplots based on the first three principal components, with individuals being colour-coded according to their assignment by STRUCTURE. Figure 2 shows that individuals belonging to the different gene pools occupy different, yet partially overlapping, areas in the DAPC scatterplot, and that the Tropical Lowland cluster is placed in an intermediate position.

### Genetic diversity and differentiation

Given the clear-cut differentiation among the NEA Flours, NEA Popcorns and the remaining regional gene pools, we analysed the levels and distribution of genetic variability among the inferred clusters. For this purpose, we computed diversity indices and pairwise allele frequency divergence estimates among groups (Table 1, Additional file 1: Table A4, Table A5).

**Table 1:** Indicators of genetic diversity within the genetic clusters inferred by STRUCTURE

	H_e_	A	R_s_	PAR	PA
NEA Flours (*N* = 344)	0.662^b^	11.1^b^	8.38^c^	0.51^c^	3
NEA Popcorns (*N* = 99)	0.383^c^	4.2^c^	4.04^d^	0.03^d^	0
Tropical Lowland (*N* = 157)	0.787^a^	17.4^a^	14.84^a^	2.24^a^	23
Andean (*N* = 275)	0.668^b^	15.5^a^	11.79^b^	0.95^b,c^	17
HM-US (*N* = 145)	0.823^a^	18.1^a^	15.87^a^	3.27^a,b^	39

*H*_*e*_ gene diversity, *A* mean number of alleles per locus, *R*_*s*_ allelic richness, *PAR* Private allelic richness, *PA* number of private alleles over all loci

Rarefaction analyses were performed with a sample size of 73. Values with different letters are significantly different from each other (*p* < 0.05, Wilcoxon signed-rank test). HM-US: Highland Mexico and US

Regardless of sample-size corrections, a consistent pattern was apparent for all the diversity estimates. The HM-US and Tropical Lowland exhibited the highest variability estimates, followed by the Andean, NEA Flours and NEA Popcorns. The HM-US also showed the highest number of private alleles in both global and pairwise comparisons (Additional file 1: Table A4).

Allele frequency divergence ranged from 0.048 (HM-US vs. Tropical Lowland) to 0.236 (HM-US vs. NEA Popcorns) (Additional file 1: Table A5). The Neighbour-joining network placed the Tropical Lowland in a central position, flanked by the HM-US and Andean on one side and the NEA Flours and NEA Popcorns on the other side, albeit with low bootstrap support (Additional file 1: Figure A5).

### *Adh2* sequence analysis

To assess whether the marked differentiation detected for the NEA groups in the SSR analyses was in agreement with the ancient structuring pattern reported by Freitas et al. [7], we examined the microsatellite allele types at the *Adh2* locus in a subset of NEA and NWA landraces from the SSR data matrix and analysed it in conjunction with previous data as described in the Methods section. To test the association between allele types and geographic regions, three groups were delimited based on the origin of the individuals, that is, Andean (west of 60° W), Middle Southern South America (MSSA, between 53°W and 60°W), and Eastern South America (ESA, east of 53°W). The MSSA region encompasses NEA and adjacent areas of Paraguay, Bolivia and western Brazil, whereas the ESA region encompasses northern, central and eastern Brazil.

**Fig. 3:**
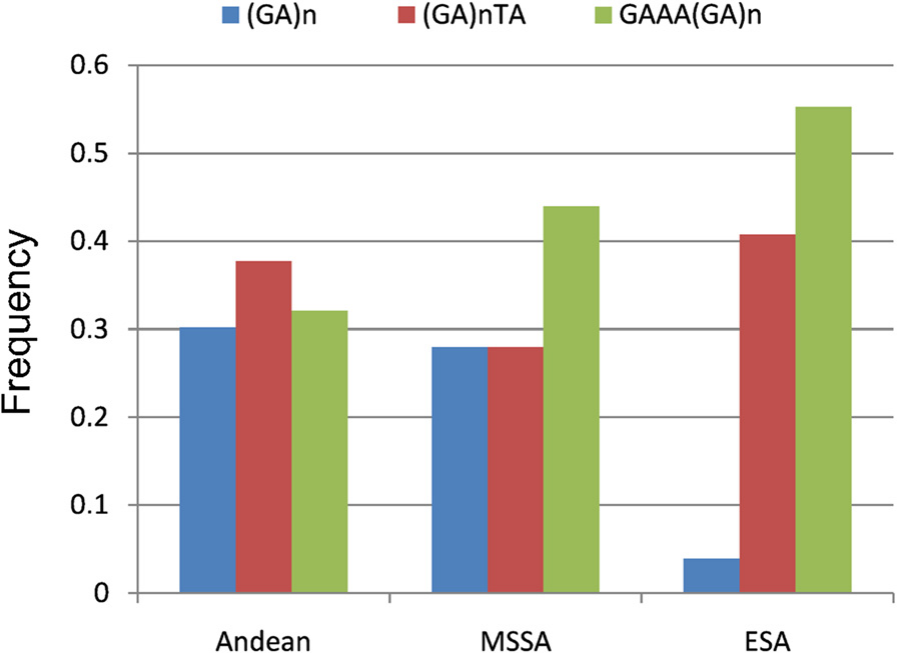
Relative abundance (by region) of *Adh2* microsatellite motifs in maize landraces from the Americas. Andean (west of 60°W), MSSA: Middle Southern South America (between 53°W and 60° W); ESA: Eastern South America (east of 53°W)

Six microsatellite allele types were recognised in the compiled *Adh2* microsatellite dataset (*N* = 497), with the most frequent being (GA)_n_, (GA)_n_TA, and GAAA(GA)_n_ (Additional file 1: Figure A6). Counts of microsatellite allele types for each region are provided in Additional file 1: Table A6. The relative abundance of these motifs in the Andean, MSSA and ESA regions is presented in Fig. 3. The distribution of allele types shows remarkable differences between the Andean and ESA regions (ML-G2 = 33.10; *p* <0.0001; d.f. = 2) and between the MSSA and ESA regions (ML-G2 = 15.26; *p* <0.0005; d.f. = 2). Conversely, differences between MSSA and the Andean region were non-significant.

### Geographic distribution modelling

The bounded geographic distribution of the genetic clusters identified here prompted us to investigate whether the observed genetic structuring was accompanied by distinct environmental requirements, especially for lowland germplasm. Habitat suitability models were obtained for four of the genetic clusters inferred by STRUCTURE, using two feature settings. Occurrence locations are given in Additional file 3: Table A7. Criterion-based model selection procedures using AICc were only applicable to the Andean and HM-US, favouring models fitted under default feature and regularisation parameters (Andean: AICc default = 2971.31; AICc hinge = 3138.87; HM-US: AICc default = 3629.28; AICc hinge = 4572.48). For the NEA Flours and Tropical Lowland, the number of parameters was higher than the number of occurrence points, thus precluding the use of AICc for model selection. Taking into account the results from model comparisons and that none of the groups showed differences in performance measures (i.e., AUCtrain and AUCtest) between default and hinge feature settings, we decided to continue our analysis based on the results with default settings. Cross-validated estimates of AUC were above 0.930 for all the groups, indicating high model discrimination ability (Table 2).

**Table 2:** Geographic distribution models of the maize clusters inferred by Bayesian analysis. Evaluation and variable importance

Genetic Cluster	k-fold cv	AUC_train_ (mean ± SD)	AUC_test_ (mean ± SD)	Variable importance (percent contribution)		
				Var1	Var2	Var3
NEA Flours	5	0.997 ± 0.001	0.994 ± 0.006	BIO3 (23.24)	BIO18 (17.26)	BIO4 (13.42)
Tropical Lowland	10	0.968 ± 0.002	0.938 ± 0.019	BIO4 (40.3)	BIO3 (22.9)	BIO16 (9.49)
Andean	10	0.992 ± 0.001	0.977 ± 0.018	Alt (35.75)	BIO4 (32.38)	BIO13 (7.04)
HM-US	10	0.968 ± 0.003	0.949 ± 0.038	BIO2 (34.49)	Alt (17.89)	BIO1 (12.39)

*cv* cross-validation, *Alt* altitude, *BIO1* Annual mean temperature, *BIO2* Mean diurnal range, *BIO3* Isothermality; *BIO4* Temperature seasonality, *BIO13* Precipitation of wettest month, *BIO16* Precipitation of wettest quarter, *BIO18* Precipitation of warmest quarter, *HM-US* Highland Mexico and US

**Fig. 4:**
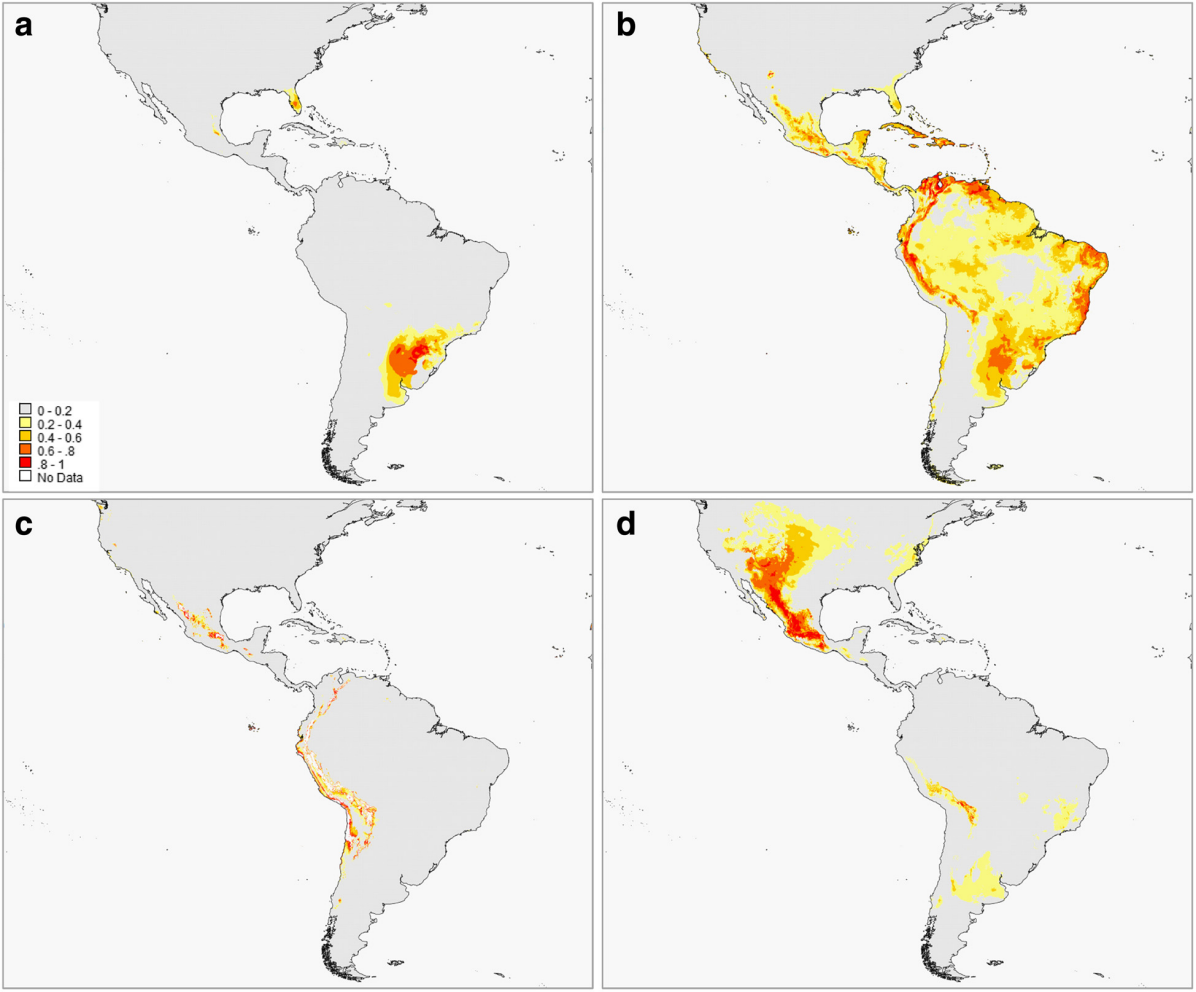
Predicted habitat suitability distributions of the genetic groups inferred for maize landraces from the Americas. **a** NEA Flours; **b** Tropical Lowland; **c** Andean; **d** Highland Mexico and US (HM-US). Warmer (red) colours represent more suitable habitats

Geographic distribution models produced by averaging cross-validation replicates are presented in Fig. 4. High values indicate a high probability of suitable conditions, intermediate values indicate conditions typical of those where the individuals are found, and low values indicate low probability of suitable conditions. The predicted distributions are in good agreement with the known cultivation areas for each group. A distinct spatial pattern is readily apparent, albeit with varying degrees of overlap among groups. The Tropical Lowland cluster showed the largest area of suitable habitats, which contrasts with the restricted predicted distribution of the NEA Flours. The Andean and HM-US also exhibited largely confined habitat suitability distributions associated with altitude.

The variables with highest average relative contribution are summarised in Table 2. The predictors with the most information not present in the other variables were: mean temperature of driest quarter (BIO9) for the NEA Flours, altitude for the Tropical Lowland, temperature seasonality (BIO4) for the Andean and precipitation of coldest quarter (BIO19) for the HM-US.

Pairwise comparison of I and D indices applied to habitat suitability distributions revealed significantly different model predictions for each group (Table 3).

**Table 3:** Comparison of habitat suitability distributions between the genetic clusters inferred for maize landraces of the Americas

	NEA Flours	Tropical lowland	Andean	HM-US
NEA Flours		0.612**	0.284**	0.414**
Tropical lowland	0.294**		0.636**	0.558**
Andean	0.127**	0.357**		0.424**
HM-US	0.183**	0.301**	0.224**	

I statistic of Warren et al. [54] (above diagonal) and Schoener’s D [55] (below diagonal). Both indices measure the similarity of habitat suitability distributions and range from 0 (no overlap) to 1 (complete overlap). HM-US: Highland Mexico and US. **p < 0.01

## Discussion

Our results reveal the occurrence of three clearly distinct gene pools in the lowlands of South America, namely: Tropical Lowland, NEA Flours and NEA Popcorns. Although Vigouroux et al. [8] recognised some sub-groups within the Tropical Lowland with a geographical distribution similar to that of the NEA groups, their importance was overlooked probably due to the sampling strategy used by these authors.

Both the Bayesian and DAPC methods show a clear separation among the NEA Flours, NEA Popcorns and the remaining clusters. Although DAPC inferred a larger optimal cluster number, the groups obtained are compatible with those from STRUCTURE since they represent subdivisions within the latter. These findings are consistent with the higher sensitivity of DAPC for detecting substructure in hierarchical models [28]. Notwithstanding these discrepancies and regardless of the presence (or not) of additional substructuring, it is clear that the genetic groups detected in the NEA are well differentiated from the other South American gene pools.

As mentioned before, the composition of the Tropical Lowland cluster obtained in our analysis is similar to that reported by Vigouroux et al. [8], including, among others, lowland accessions from southwestern Mexico, which has been proposed as the centre of maize domestication. The central position of this assemblage in both the DAPC scatterplot and the Neighbour-joining network is consistent with the ancestral condition of the Tropical Lowland germplasm. Moreover, this group shows the lowest value of the genetic drift parameter (F), thus supporting its ancestral condition. In the present work, the Andean complex emerged as a clearly separate entity, in agreement with several previous studies [1, 6, 9, 10]. According to van Heerwaarden et al. [12] the Andean germplasm was the most divergent group from a common hypothetical ancestor, i.e. had the largest estimate of the drift parameter, but our results indicate an even greater degree of divergence for the NEA Flours and the NEA Popcorns, once again highlighting their uniqueness. In contrast with the results of Vigouroux et al. [8], our analyses failed to discriminate between Highland Mexico and US landraces, which were thus assigned to a single complex. This could be partly due to a decrease in the number of markers, and also to the inclusion of new individuals belonging to two well-differentiated groups (i.e. NEA Flours and NEA Popcorns). However, this lack of resolution is compatible with the similarities previously described by Vigouroux et al. [8], who suggested that the landraces of northern US were derived from those of the southwestern US, which in turn were derived from those of northern Mexico.

The levels of diversity constitute an important factor in interpreting the high differentiation of the NEA groups. If these would have derived from any of the major groups in relatively recent times and then subjected to isolation by cultural or environmental factors, one would expect the diversity levels to be low and the allelic variants to be a subset of the original gene pool. Our analysis of diversity indices revealed that the variability levels of the NEA Flours were similar to those of the Andean group, whereas the NEA Popcorns had the lowest estimates (Table 1). Moreover, the NEA Flours also showed private alleles when compared to the other groups -though in small number- evidencing that the divergence found here is not only based on differences in the distribution of allelic frequencies. Interestingly, the highest diversity indices, including the number of private alleles, corresponded to the HM-US and Tropical Lowland groups, with the former having slightly higher values. Although differences were not statistically significant, these results are consistent with recent studies suggesting that maize landraces from the Mexican highlands received a substantial genetic contribution from the teosinte *Zea mays* ssp. *mexicana* [12]. In summary, the levels of genetic diversity found for NEA Flours do not coincide with those expected under a severe bottle-neck scenario followed by isolation; in contrast, the NEA Popcorns showed a remarkably low variability.

The frequency distribution analysis of the *Adh2* microsatellite allele types considered here revealed that statistically significant differences were found between ESA and MSSA, and between ESA and Andean regions. Since most of the data were collected from the literature, we could not establish a direct link between the geographic origin of all the individuals included in the analysis and their membership to any of the genetic groups inferred from our SSR analysis. However, the differences in the distribution of *Adh2* microsatellite motifs between ESA and MSSA provide additional evidence favouring the existence of two distinct genetic groups in the lowlands of South America, with NEA Flours and NEA Popcorns sampling localities being contained within the boundaries of the MSSA category. On the other hand, the homogeneity found between Andean and MSSA is in agreement with the view of previous authors that Andean germplasm had an influence on the landraces of middle South America [6, 8, 10].

Local adaptation has played a key role in the dispersal and adoption of the different maize landraces which are currently cultivated in South America. Native landraces are maintained by local small-scale producers using traditional agro-technologies, with yield largely depending on weather conditions. On this basis, the knowledge of crop environmental requirements across different areas, together with a comprehensive genetic characterisation, may greatly contribute to elucidate the mechanisms underlying adaptation patterns at a local scale. Our work is the first one to investigate the relationship between genetic groups and environmental distribution models for South American maize. Distribution modelling studies focused on maize have been conducted at the landrace level [16, 17]. It is well-known, however, that the name given to a landrace does not always correspond to its genetic constitution, and that different entities assigned to the same landrace may differ more from each other than from the entities of other landraces. In an attempt to overcome these difficulties, we modelled the distribution of the genetic groups regardless of their designation because we assumed that the genetic composition is more informative on the adaptation to environmental conditions. Besides the evidence of differentiation provided by the analyses of SSR and *Adh2* microsatellite variability, the inferred genetic groups from the lowlands of South America, i.e. Tropical Lowland and NEA Flours, also showed significantly different habitat suitability models, not only between each other but also with the other gene pools (Table 3).

The two groups from the lowlands are strongly influenced by variables related to temperature (isothermality and temperature seasonality), though they seem to be affected differently by the rainfall regime, with a greater impact on NEA Flours. Altitude appears as a determinant factor for HM-US and Andean (Table 2), but with different maximum gain values (about 2500 and 3500 m, respectively). In agreement with our findings, recent studies of adaptation to high elevation in maize revealed that Highland Mexico and Andean landraces have largely distinct gene sets involved in highland adaptation [56]. On the other hand, it is worthy to mention that only the model of HM-US shows an important contribution of the variable mean diurnal range, probably because this group includes numerous temperate accessions.

Like in our study, Hufford et al. [16] found that temperature seasonality is the most important variable for the teosintes and four indigenous maize landraces of Mexico. However, despite the fact that their landraces would probably be included in one or more of the groups inferred here, in general, they obtained different importance rankings for the variables. This could be related to the grouping criterion (i.e. landrace assignment vs. genetic structure), and to differences in the geographic scale considered. Indeed, it has been proposed that climatic variables are major limiting factors at large spatial scales, whereas the effect of climate is often masked by responses to local environmental variables such as soil, terrain, and habitat type at finer spatial scales [57].

In addition to our results of genetic and distribution modelling, diverse sources of evidence support the existence of a particular maize group from lowland middle South America. Most of the landraces of NEA Flours and all of the landraces of NEA Popcorns were collected from the Guaraní indigenous communities settled in the province of Misiones, Argentina. In contrast, NEA landraces collected outside these communities and currently maintained in the Germplasm Bank of INTA Pergamino, appeared to be greatly influenced by the Tropical Lowland gene pool (Fig. 1). In accordance with our findings, McClintock et al. [6] identified a South American complex named “Central Region group” of uncertain origin and age. The landraces of this group showed distinctive chromosome features contrasting markedly with landraces occurring in the vicinity; they were cultivated by the Guaraní and Kaingang communities principally distributed in Northeastern Argentina, Paraguay, southern Bolivia and southwestern Brazil. Moreover, the germplasm cultivated by the Guaraní people was included into the category “indigenous landraces” by Paterniani & Goodman [58], who recognised four major types: Avatí morotí, a floury yellow corn (the most abundant type), Avatí tupí or Cristal, a white Flint, and two popcorns, namely the round kernel type and the pointed kernel type. These authors assumed that popcorn maize was only cultivated in the region by the Guaraní people, whereas the Avatí morotí type was an ancient group which spread earlier, at a time when there were few maize landrace groups. In this context, it is reasonable to hypothesize that the landraces within the NEA Flours, which are locally known as Avatí (e.g. Avatí morotí, Avatí pará, Avatí yui) [59], correspond to either the Central Region or Avatí Morotí groups. Likewise, the remarkable genetic differentiation and the low variability levels among the NEA Popcorns coincide with the classification of Sanchez et al. [60] for the “Guaraní Popcorns” group based on morphological and isoenzymatic data; this also suggests a possible correspondence between both groups. Further study is needed to elucidate the precise origin of the NEA germplasm, as well as the route and date of their introduction.

## Conclusions

Different authors have proposed middle South America as a contact region where the Andean landraces interbred with those of the eastern coast of South America [8, 11]. However, our data indicate that it is only a partial description of maize dynamics in middle South America. We believe that this region may have played a far more important role in the structuring of genetic variation since it harbours a unique, locally adapted gene pool. Here, we highlight its distinctiveness for the first time, and provide relevant information for the conservation and utilization of these genetic resources, as well as for a better understanding of environment-genotype associations in the context of maize population structure and historical processes.

## Additional files

Additional file 1: Figure A1. Sampling localities of the individuals included in this study. **Figure A2**. Evaluation of STRUCTURE outputs. **Figure A3**. Sampling intensity and population structure. **Figure A4**. Discriminant Analysis of Principal Components (DAPC). **Figure A5**. Neighbour-joining network based on F_ST_ coefficients between the genetic clusters of maize landraces inferred by STRUCTURE. **Figure A6**. Frequency of the *Adh2* microsatellite allele types in maize landraces from the Americas (*N* = 497). **Table A1**. Maize landraces from Northeastern Argentina genotyped for the present study. **Table A3**. *Adh2* microsatellite allele types present in the landraces sequenced in this study. MSSA: Middle Southern South America. **Table A4**. Pair-wise comparison of private alleles between the genetic clusters inferred by STRUCTURE. **Table A5**. Differentiation among maize genetic clusters. **Table A6**. *Adh2* microsatellite allele type counts *per* region including archaeological specimens, primitive and historic landraces (*N* = 497). Data from this study, Goloubinoff et al. [42], Freitas et al. [7] and Grimaldo Giraldo [18]. (DOCX 3582 kb) Additional file 2: Table A2. Compiled SSR data matrix (Excel). (XLSX 110 kb) Additional file 3: Table A7. Occurrence locations used for geographic distribution modelling. (XLSX 18 kb)

## Abbreviations

AICc: Sample-size corrected Akaike’s Information Criteria
AUC: Area under the receiver operating characteristic curve
DAPC: Discriminant Analysis of Principal Components
ESA: Eastern South America
HM-US: Highland Mexico and United States
MSSA: Middle Southern South America
NEA: Northeastern Argentina
NWA: Northwestern Argentina
SSR: Simple Sequence Repeat.
