# Supplementary material for: Dissecting maize diversity in lowland South America: genetic structure and geographic distribution models
Source: BMC Plant Biol. 2016 Aug 26;16(1):186. doi: 10.1186/s12870-016-0874-5 (PMC5000442; doi:10.1186/s12870-016-0874-5)
Supplement: Additional file 1: Figure A1. — Sampling localities of the individuals included in this study. Figure A2. Evaluation of STRUCTURE outputs. Figure A3. Sampling intensity and population structure. Figure A4. Discriminant Analysis of Principal Components (DAPC). Figure A5. Neighbour-joining network based on FST coefficients between the genetic clusters of maize landraces inferred by STRUCTURE. Figure A6. Frequency of the Adh2 microsatellite allele types in maize landraces from the Americas (N = 497). Table A1. Maize landraces from Northeastern Argentina genotyped for the present study. Table A3. Adh2 microsatellite allele types present in the landraces sequenced in this study. MSSA: Middle Southern South America. Table A4. Pair-wise comparison of private alleles between the genetic clusters inferred by STRUCTURE. Table A5. Differentiation among maize genetic clusters. Table A6. Adh2 microsatellite allele type counts per region including archaeological specimens, primitive and historic landraces (N = 497). Data from this study, Goloubinoff et al. [42], Freitas et al. [7] and Grimaldo Giraldo [18]. (DOCX 3582 kb) [file 12870_2016_874_MOESM1_ESM.docx]

### **Fig. A1.** Sampling localities of the individuals included in this study. Misiones Province: 1*, Colonia Mado Delicia; 2*, Aldea Alecrín; 3*, Aldea Pozo Azul; 4*, Piñalito; 5, Santo Pipó; 6, Campo Las Monjas; 7, Tobuna; 8, Bernardo de Irigoyen. Chaco Province: 9, La Eduvigis; 10, Lapachito; 11, Pte. Roque Sáenz Peña; 12, Tres Isletas; 13, Castelli; Corrientes Province: 14, Corrientes; 15, El Sombrero; 16, Ifrán; 17, Perrugorría; 18, Colonia Libertad; 19, Paraje Tacuaba; Entre Rios Province: 20, Colonia Tunas; 21, Colonia Razzetta; 22, Colonia Sauce; 23, Colonia Cricecita; 24, Colonia Nueva; Formosa Province: 25, Gral. Manuel Belgrano; 26, Colonia Bañaderos; 27, Misión Tacaaglé; 28, El Olvido. *Guaraní indigenous communities.


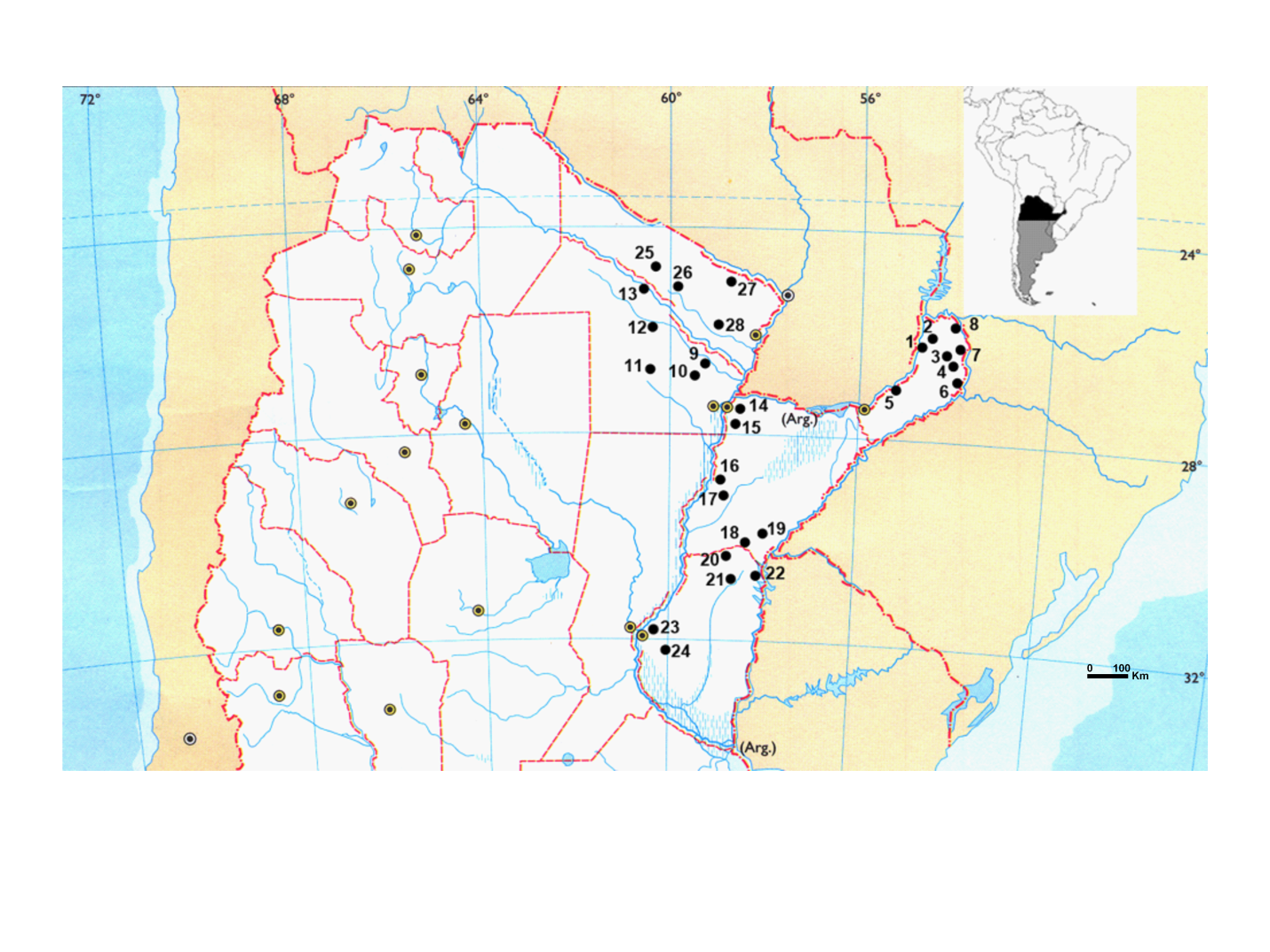


### **Fig. A2.** Evaluation of STRUCTURE outputs. (a) Mean Ln P (D) over 10 runs for each K value. (b) Second order rate of change in the likelihood of *K* (∆K).


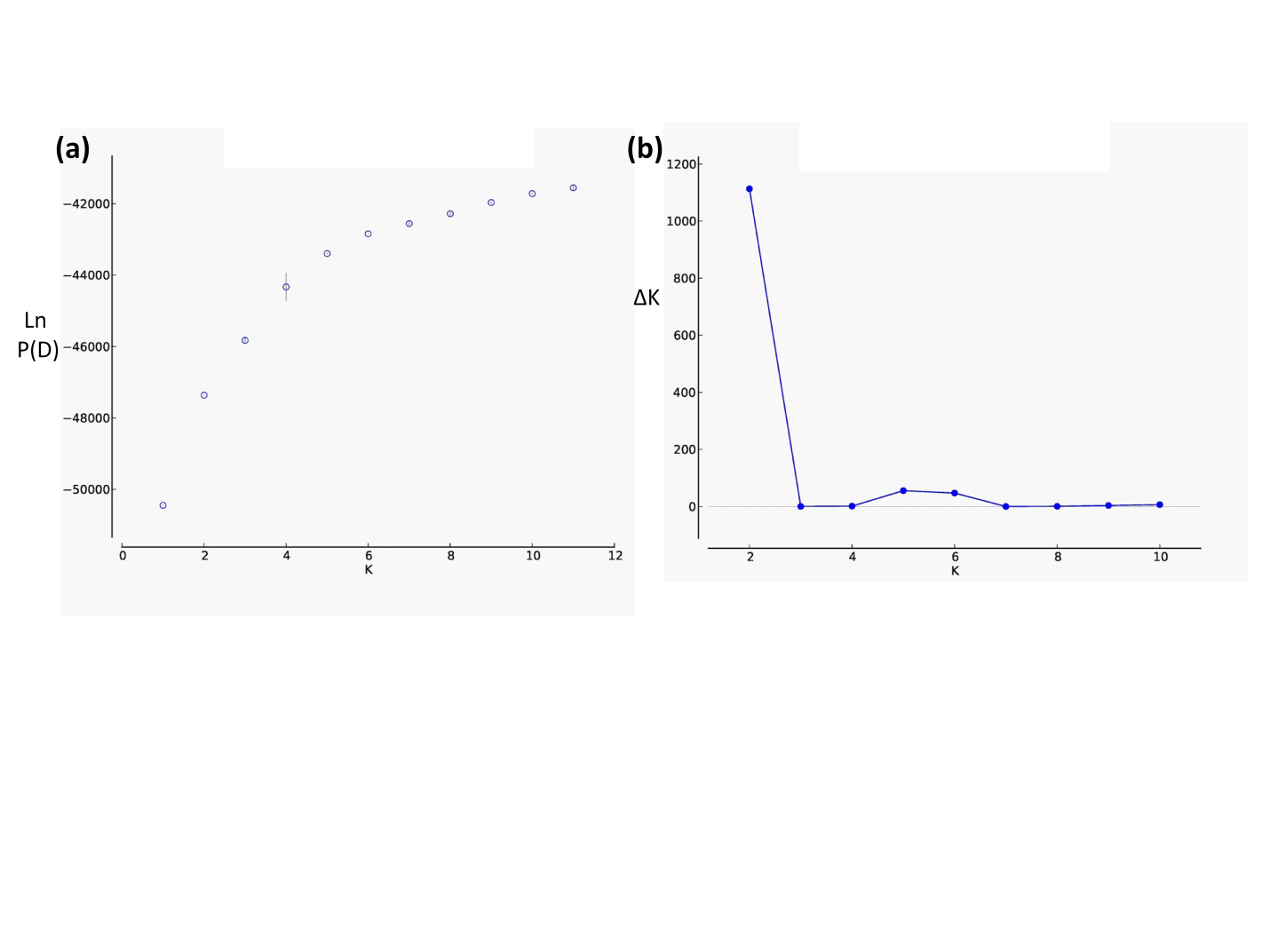


### **Fig. A3.** Sampling intensity and population structure. STRUCTURE output obtained from a SSR data matrix in which the number of individuals from the NEA Flours and NEA Popcorns groups was reduced to 50. ^1^ Data from Vigouroux et al. (2008); ^2^ data from Lia et al. (2009); ^3^ data from Bracco et al. (2012); ^4^ data from this study.

**
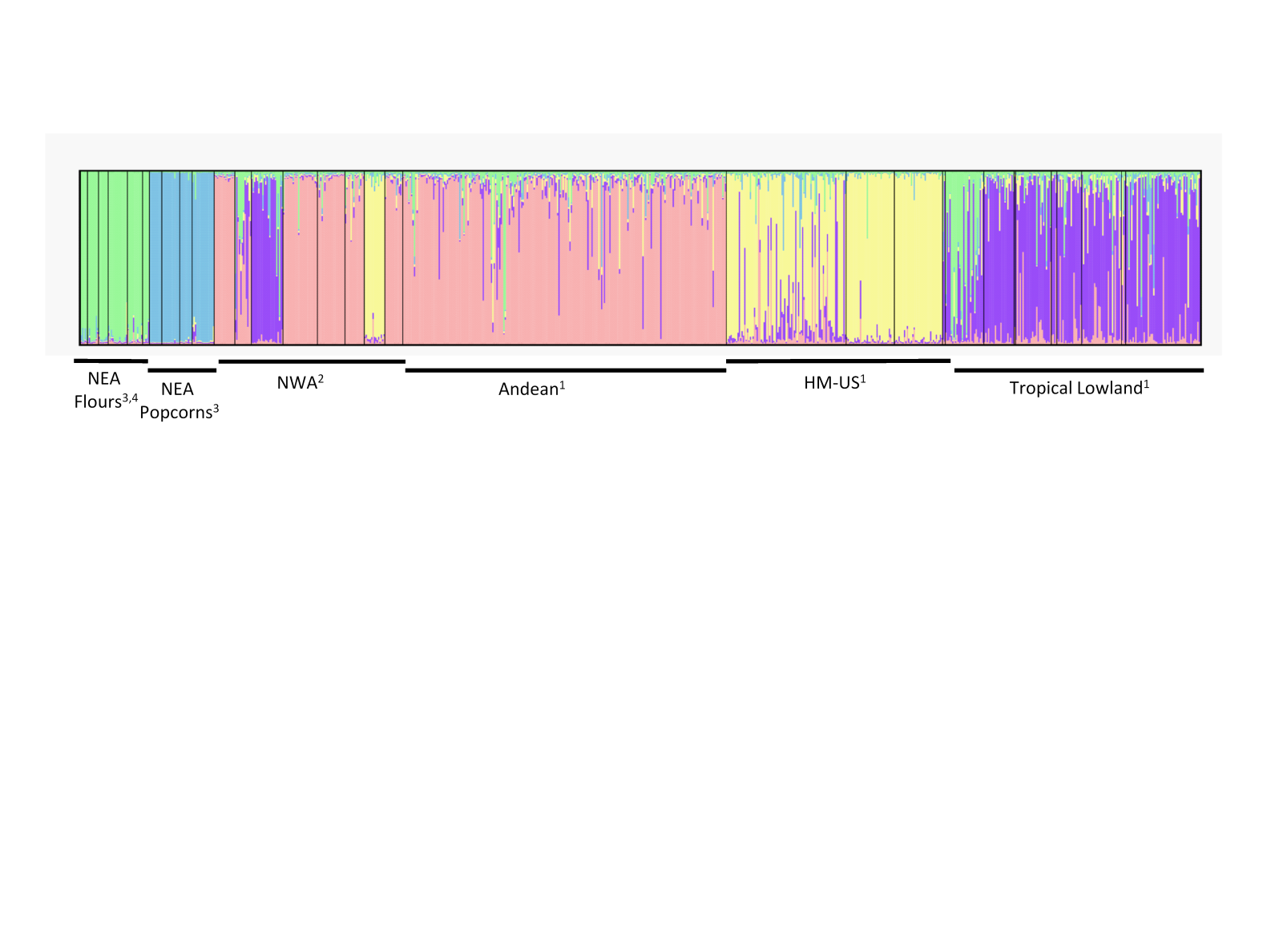
**

###
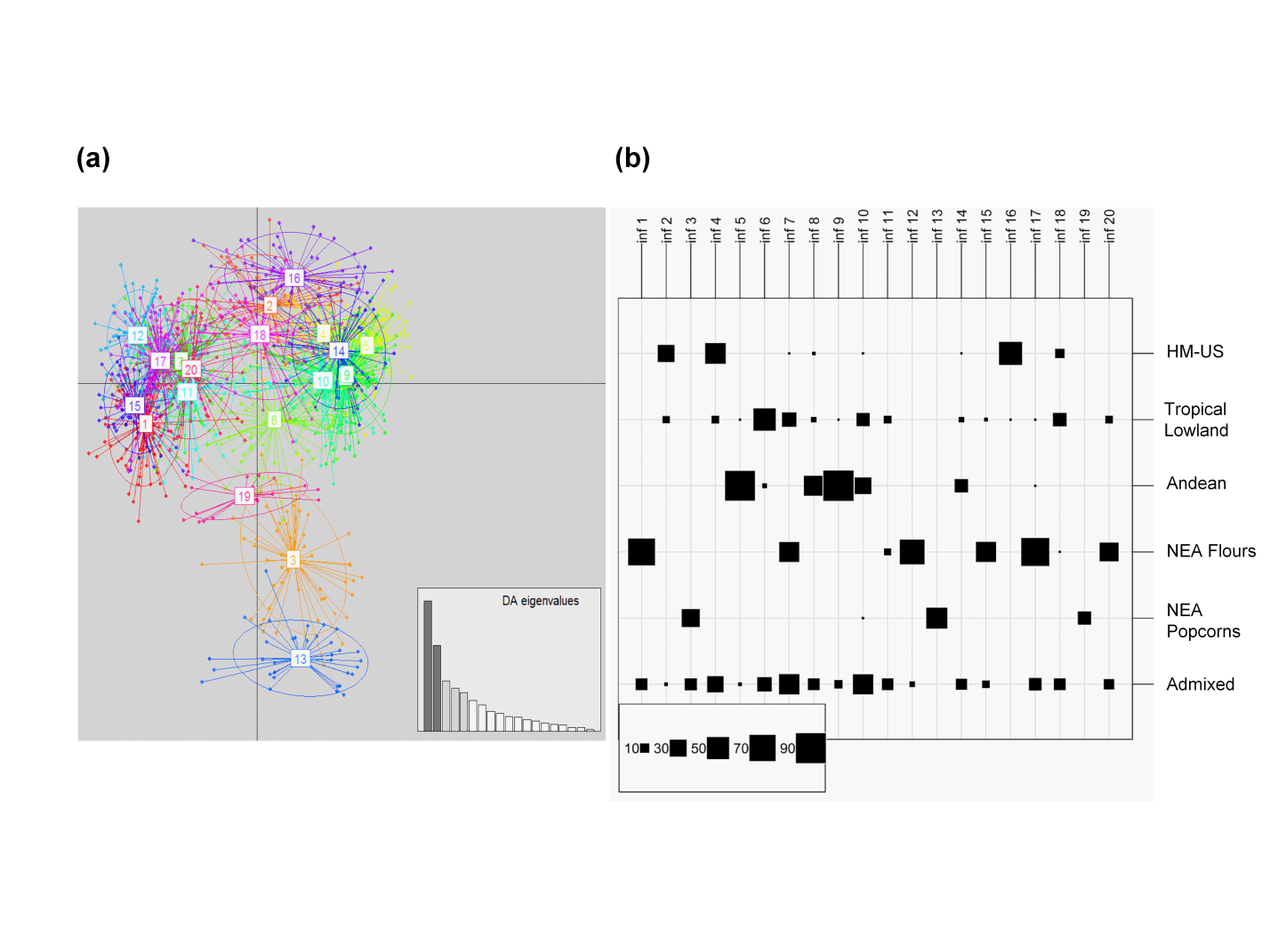
**Fig. A4. (**a) Scatterplot of the Discriminant Analysis of Principal Components (DAPC). Dots represent individual samples. (b) Cluster assignment obtained from the k-means algorithm.

### **Fig. A5.** Neighbour-joining network based on allele frequency divergence estimates between the genetic clusters of maize landraces inferred by STRUCTURE. Bootstrap values are given above branches.


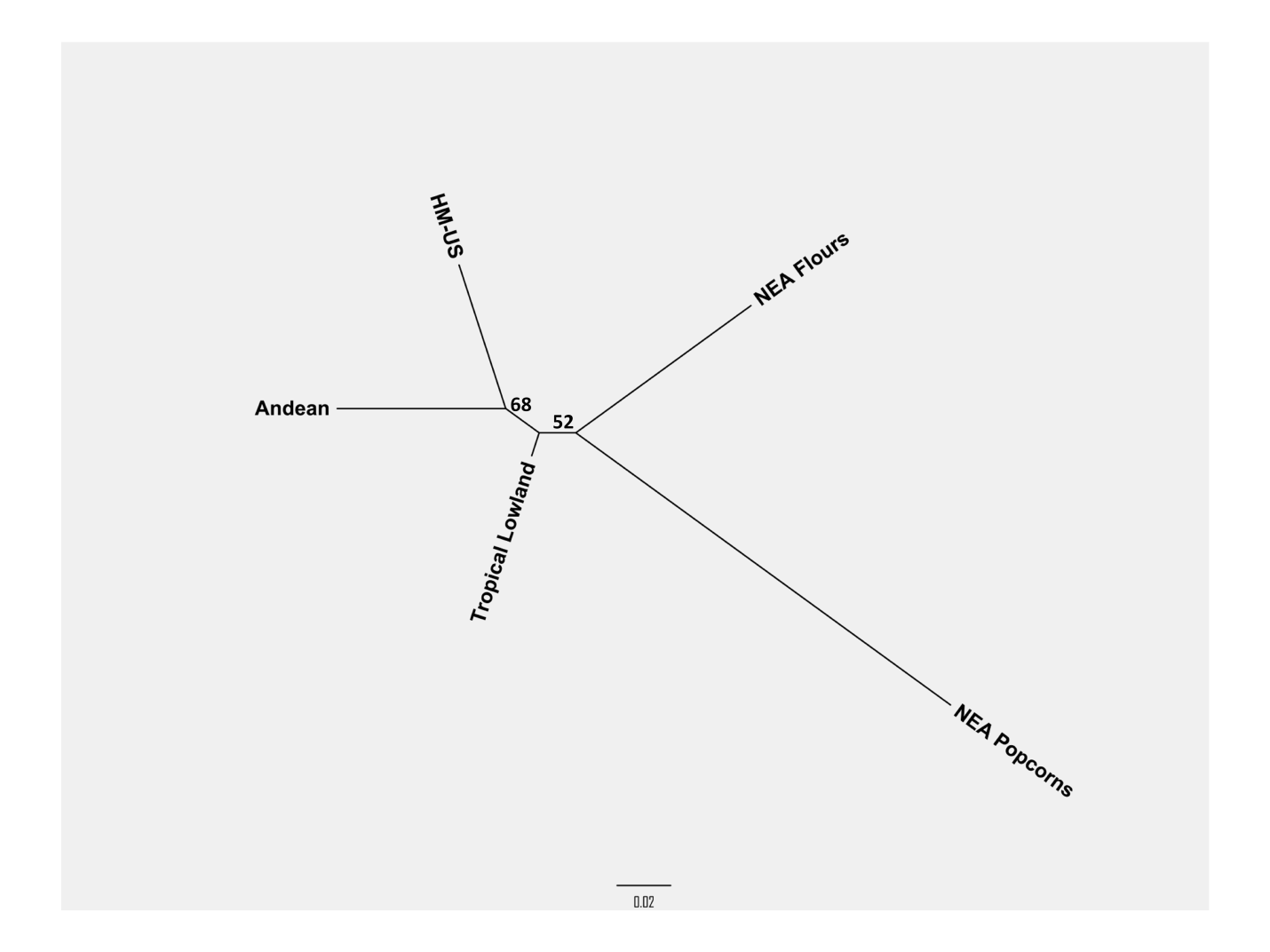


### **Fig. A6.** Frequency of the *Adh2* microsatellite allele types in maize landraces from the Americas (N=497).

**
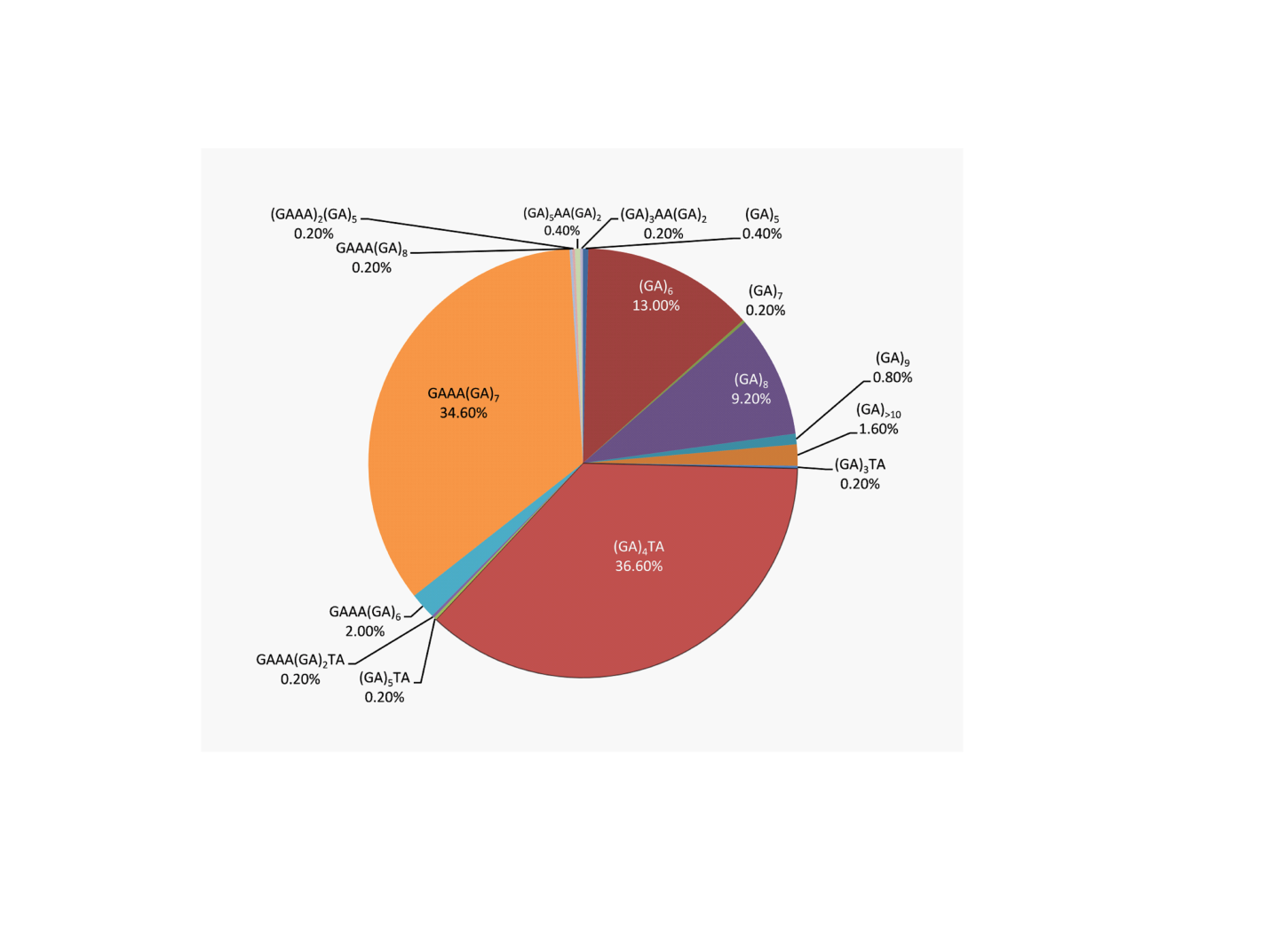
**

### **Table A1.** Maize landraces from Northeastern Argentina genotyped for the present study.

| **Landrace** | **Accession ^a^** | **Collection site^b^** | **Sample size** |
| --- | --- | --- | --- |
| Amarillo ancho | VAV6611 | Pozo Azul, Dpto. San Pedro **(3)** | 24 |
| Amarillo angosto | VAV6593 | Aldea Alecrín, Dpto. Eldorado **(2)** | 28 |
|  | VAV6591 | Pozo Azul, Dpto. San Pedro **(3)** | 16 |
| Avatí morotí | ARZM04015 | Paraje Tacuaba, Monte Caseros, Prov. Corrientes **(19)**. | 4 |
|  | ARZM04025 | Perugorría, Curuzú Cuatiá, Prov. Corrientes **(17)**. | 5 |
|  | ARZM04026; ARZM04029 | Ifrán, Goya, Prov. Corrientes **(16)**. | 5; 5 |
|  | ARZM05069 | Campo Las Monjas km 300, Prov. Misiones **(6)**. | 4 |
|  | ARZM05077 | Bernardo de Irigoyen, Gral. Belgrano, Prov. Misiones **(8)**. | 3 |
|  | ARZM05118 | Santo Pipó, San Ignacio, Prov. Misiones **(5)**. | 4 |
|  | ARZM05067 | Campo Las Monjas km 300, Prov Misiones **(6)**. | 5 |
|  | ARZM06067 | Tres Isletas, Maipú, Prov. Chaco **(12)**. | 7 |
|  | ARZM06109 | Lapachito, Gral. Donovan, Prov. Chaco **(10)**. | 5 |
|  | ARZM07094 | El Olvido, Pirané, Prov. Formosa **(28)**. | 3 |
| Calchaquí | ARZM04060 | Corrientes, Corrientes, Prov. Corrientes **(14)**. | 5 |
|  | ARZM03036 | Colonia Nueva, Feliciano, Prov. Entre Ríos **(24)**. | 4 |
| Camelia | ARZM04018 | Colonia Libertad, Monte Caseros, Prov. Corrientes **(18)**. | 5 |
| Canario de Formosa | ARZM04063 | El sombrero, Corrientes, Prov. Corrientes **(15)**. | 4 |
|  | ARZM07058 | Gral. Manuel Belgrano, Patiño, Prov. Formosa **(25)**. | 1 |
|  | ARZM07061 | Misión Tacaaglé, Pilagás, Prov. Formosa **(27)**. | 2 |
| Cristalino amarillo anaranjado | ARZM06002 | La Eduvigis, Libertador San Martín, Prov. Chaco **(9)**. | 3 |
|  | ARZM03021 | Colonia Razzetta, Federación, Prov. Entre Ríos **(21)**. | 5 |
|  | ARZM03055 | Colonia 21 Cricecita, Nogoyá, Prov. Entre Ríos **(23)**. | 5 |
| Cristalino colorado | ARZM06050 | Pte. R. Sáenz Peña, Cte. Fernández, Prov. Chaco **(11)**. | 2 |
| Dentado blanco | ARZM06076 | Castelli, Gral. Martín de Güemes, Prov. Chaco **(13)**. | 5 |
|  | ARZM07125 | Colonia Bañaderos, Patiño, Prov. Formosa **(26)**. | 3 |
|  | ARZM03011 | Colonia Sauce, Federación, Prov. Entre Ríos **(22)**. | 5 |
|  | ARZM03030 | Colonia Tunas, Feliciano, Prov. Entre Ríos **(20)**. | 5 |
| Rosado | VAV6603; VAV6602b | Pozo Azul, Dpto. San Pedro **(3)** | 20 |
| Tupí amarillo | VAV6619 | Colonia Mado Delicia, Dpto. Eldorado **(1)** | 16 |
|  | VAV6608 | Piñalito, Dpto. San Pedro **(4)** | 20 |
| Venezolano | ARZM05074 | Tobuna, San Pedro, Prov. Misiones **(7)**. | 4 |
| ^a^ Accessions designated VAV were collected by the authors in 2005 and are deposited at the ‘Laboratorio de Recursos Genéticos Vegetales N.I. Vavilov’ (VAV), Facultad de Agronomía, Universidad de Buenos Aires, Argentina. Accessions designated ARZM were provided by the Germplasm Bank “Estación Experimental Agropecuaria INTA-Pergamino”, Argentina. These accessions correspond to the original collections conducted during 1977-1978. ^b^ Bold numbers in parentheses correspond to the geographical locations given in Figure S1. | | | |

### **Table A3.** *Adh2* microsatellite allele types present in the landraces sequenced in this study. MSSA: Middle Southern South America.

| **Allele type** | **Landrace** | **Accession**  **(individual)*** | **Region** |
| --- | --- | --- | --- |
| **(GA)_n_** |  |  |  |
| (GA)_5_ | Altiplano | VAV6473 (D34) | Andean |
| (GA)_8_ | Tupí amarillo | VAV6619 (I324) | MSSA |
|  | Blanco ancho | VAV6586 (I4) | MSSA |
|  | Azul de Misiones | VAV6597 (I177) | MSSA |
|  | Avatí morotí | ARZM7094 (474) | MSSA |
|  | Camelia | ARZM4018 (363) | MSSA |
|  | Amarillo chico | VAV6484 (C180) | Andean |
| **(GA)_n_TA** |  |  |  |
| (GA)4 | Blanco angosto | VAV6599 (VII72) | MSSA |
|  | Altiplano | VAV6167 (K224) | Andean |
|  | Colorado de Misiones | VAV6604 (I166) | MSSA |
|  | Rosado | VAV6603 (I233) | MSSA |
|  | Variegado | VAV6585 (I201) | MSSA |
|  | Pororó grande | VAV6614 (I224p) | MSSA |
|  | Avatí morotí | ARZM5118 (449) | MSSA |
|  | Avatí morotí tí | ARZM5067 (436) | MSSA |
|  | Avatí morotí | ARZM5077 (429) | MSSA |
|  | Canario de Formosa | ARZM7061 (484) | MSSA |
| **GAAA(GA)_n_** |  |  |  |
| GAAA(GA)_7_ | Blanco angosto | VAV6610 (IV87) | MSSA |
|  | Pipoca colorado | VAV6596 (I52p) | MSSA |
|  | Pipoca Amarillo | VAV6605 (III103p) | MSSA |
|  | Pipoca Colorado | VAV6618 (I134p) | MSSA |
|  | Overo | VAV6601 (II132) | MSSA |
|  | Amarillo grande | VAV6480 (A152) | Andean |
| (GA)_2_AA(GA)_3_ | Amarillo chico | VAV6476 (D90) | Andean |

*Individual name as designated in the SSR data matrix.

### **Table A4.** Pair-wise comparison of private alleles between the genetic clusters inferred by STRUCTURE.

| **Present** | **Absent** | **NEA Flours** | | **NEA Popcorns** | **Tropical Lowland** | **Andean** | **HM-US** |
| --- | --- | --- | --- | --- | --- | --- | --- |
|  | |  | |  |  |  |  |
| **NEA Flours** | | |  | 73 | 11 | 15 | 18 |
| **NEA Pocorns** | | | 4 |  | 2 | 4 | 2 |
| **Tropical Lowland** | | | 74 | 134 |  | 49 | 42 |
| **Andean** | | | 59 | 117 | 30 |  | 33 |
| **HM-US** | | | 88 | 141 | 49 | 59 |  |
|  | |  | |  |  |  |  |
| The total number of alleles across samples and loci was 247. HM-US: Highland Mexico and US. | | | | | | | |

### **Table A5.** Differentiation among maize genetic clusters. Allele frequency divergence estimated by STRUCTURE (below diagonal).

|  | **NEA Flours** | **NEA popcorn** | **Tropical Lowland** | **Andean** | **HM-US** |
| --- | --- | --- | --- | --- | --- |
| **NEA Flours** |  |  |  |  |  |
| **NEA Popcorns** | 0.2040 |  |  |  |  |
| **Tropical Lowland** | 0.0661 | 0.1564 |  |  |  |
| **Andean** | 0.1585 | 0.1837 | 0.0705 |  |  |
| **HM-US** | 0.1251 | 0.2359 | 0.0488 | 0.0870 |  |

### **Table A6.** *Adh2* microsatellite allele type counts *per* region including archaeological specimens, primitive and historic landraces (N=497). Data from this study, Goloubinoff *et al.* (1993), Freitas *et al.* (2003) and Grimaldo Giraldo (2011).

| Allele type | Region | | |
| --- | --- | --- | --- |
|  | Andean | Middle southern  South America | Eastern South  America |
| (GA)n | 112 | 14 | 3 |
| (GA)nTA | 140 | 14 | 31 |
| GAAA(GA)n | 119 | 22 | 42 |
